# Digital behaviour change interventions to increase vegetable intake in adults: a systematic review

**DOI:** 10.1186/s12966-023-01439-9

**Published:** 2023-03-27

**Authors:** Katherine M. Livingstone, Jonathan C. Rawstorn, Stephanie R. Partridge, Stephanie L. Godrich, Sarah A. McNaughton, Gilly A. Hendrie, Lauren C. Blekkenhorst, Ralph Maddison, Yuxin Zhang, Scott Barnett, John C. Mathers, Maria Packard, Laura Alston

**Affiliations:** 1 Institute for Physical Activity and Nutrition, School of Exercise and Nutrition Sciences, Deakin University, Melbourne Burwood Campus, 221 Burwood Highway, VIC 3125 Melbourne, Australia; 2 Institute for Physical Activity and Nutrition (IPAN), School of Exercise and Nutrition Sciences, Deakin University, Geelong, VIC 3220 Australia; 3 Engagement and Co-Design Research Hub, School of Health Sciences, Faculty of Medicine and Health, The University of Sydney, Sydney, NSW Australia; 4 School of Medical and Health Sciences, Nutrition and Health Innovation Research Institute, Edith Cowan University, Bunbury, WA 6230 Australia; 5 Human Health Program, Health & Biosecurity, CSIRO, Adelaide, SA 5000 Australia; 6 School of Medical and Health Sciences, Nutrition and Health Innovation Research Institute, Edith Cowan University, Perth, Australia; 7 Applied Artificial Intelligence Institute (A²I²), Deakin University, Geelong, Australia; 8 Human Nutrition & Exercise Research Centre, Centre for Healthier Lives, Population Health Sciences Institute, Newcastle University, Newcastle Upon Tyne, NE2 4HH UK; 9 The National Heart Foundation of Australia, Melbourne, VIC 3000 Australia; 10 Deakin Rural Health, School of Medicine, Faculty of Health, Deakin University, Warrnambool, Australia; 11 The Global Obesity Centre, Institute for Health Transformation, Faculty of Health, Deakin University, 1 Gheringhap Street, Geelong, VIC 3220 Australia

**Keywords:** Digital, Behaviour change, Intervention, Vegetable intake, Co-design, Personalisation, Adults, Systematic review

## Abstract

**Background:**

Digital interventions may help address low vegetable intake in adults, however there is limited understanding of the features that make them effective. We systematically reviewed digital interventions to increase vegetable intake to 1) describe the effectiveness of the interventions; 2) examine links between effectiveness and use of co-design, personalisation, behavioural theories, and/or a policy framework; and 3) identify other features that contribute to effectiveness.

**Methods:**

A systematic search strategy was used to identify eligible studies from MEDLINE, Embase, PsycINFO, Scopus, CINAHL, Cochrane Library, INFORMIT, IEEE Xplore and Clinical Trial Registries, published between January 2000 and August 2022. Digital interventions to increase vegetable intake were included, with effective interventions identified based on statistically significant improvement in vegetable intake. To identify policy-action gaps, studies were mapped across the three domains of the NOURISHING framework (i.e., behaviour change communication, food environment, and food system). Risk of bias was assessed using Cochrane tools for randomized, cluster randomized and non-randomized trials.

**Results:**

Of the 1,347 records identified, 30 studies were included. Risk of bias was high or serious in most studies (*n* = 25/30; 83%). Approximately one quarter of the included interventions (*n* = 8) were effective at improving vegetable intake. While the features of effective and ineffective interventions were similar, embedding of behaviour change theories (89% vs 61%) and inclusion of stakeholders in the design of the intervention (50% vs 38%) were more common among effective interventions. Only one (ineffective) intervention used true co-design. Although fewer effective interventions included personalisation (67% vs 81%), the degree of personalisation varied considerably between studies. All interventions mapped across the NOURISHING framework behaviour change communication domain, with one ineffective intervention also mapping across the food environment domain.

**Conclusion:**

Few digital interventions identified in this review were effective for increasing vegetable intake. Embedding behaviour change theories and involving stakeholders in intervention design may increase the likelihood of success. The under-utilisation of comprehensive co-design methods presents an opportunity to ensure that personalisation approaches better meet the needs of target populations. Moreover, future digital interventions should address both behaviour change and food environment influences on vegetable intake.

**Supplementary Information:**

The online version contains supplementary material available at 10.1186/s12966-023-01439-9.

## Background

Low vegetable and legume consumption is a leading modifiable risk factor for non-communicable diseases globally [1, 2], accounting for over 2% of global deaths in 2017 [1]. International guidelines for vegetable intake recommend at least 3 serves/day (≥ 240 g/day) [3]. However, nationally representative survey data from 162 countries found that, in 2020, an average of 88% of the populations of these countries had an inadequate vegetable intake [4].

Interventions designed to address low vegetable intake often target low fruit intake simultaneously [5]; however, this is more likely to increase fruit intake than vegetable intake [6]. This is largely attributable to interventions not addressing barriers to vegetable intake, which are distinct from those of fruit intake, including lower palatability, lack of cooking confidence, and perceived higher cost and time to purchase, prepare and cook vegetable-rich meals [6–11]. Interventions that specifically focus on vegetables show promise, but are often setting-specific and delivered face-to-face, such as a workplace interventions [12]. While setting-specificity may be an important component of some personalisation approaches, more scalable approaches are needed to ensure interventions can serve large populations across a wide range of settings [13–15].

As an estimated 66% of people globally have access to the internet [16], digital interventions provide an accessible delivery model for increasing vegetable intake in adults [10, 11]. Furthermore, digital interventions are well aligned with the global drive to utilise digital technologies to improve health [17]. For example, 55% of European citizens aged 16–74 reported that they had sought online health information [18], and 88% of Australians reported wanting to access their health information digitally [19]. However, while there is some evidence that digital interventions increase fruit and vegetable intake [20], the effectiveness of digital interventions to increase vegetable intake alone is unclear.

Digital interventions offer the ability to personalise content and delivery to the needs and preferences of the user. Although evidence from randomised controlled trials (RCTs) suggest that personalised dietary advice motivates greater improvement in dietary intake than generalised dietary advice [21], personalisation of digital interventions alone may not be sufficient to increase vegetable intake. To help ensure dietary interventions meet the needs of the user, interventions are increasingly being designed with stakeholders, i.e., using co-design practices [22].

Co-design practices involve the lived experiences of the users, and individuals with technical expertise or service providers in the design process [23]. Research suggests that the use of co-design may help improve consumer engagement and satisfaction with a digital intervention by ensuring it meets their needs [23–25]. However, there is limited understanding of whether existing digital interventions to increase vegetable intake have used co-design methods or whether the use of co-design contributes to effectiveness.

Mediators of behaviour change, including knowledge of, attitudes towards, and skills in using vegetables, can be targeted in digital interventions to meet the needs of the user [26, 27]. However, achieving higher vegetable intake is also dependent on complex interactions between individual- and environmental-level influences, such as self-efficacy or access to affordable and healthy foods, which require specific policy actions [7, 8]. The NOURISHING framework [28], which maps interventions according to their alignment with policy actions related to behaviour change communications, the food environment or the food system, is a useful framework for considering such approaches. By mapping across each of these domains, gaps, and opportunities for policy actions for achieving behaviour change can be identified and targeted by digital interventions. Therefore, we aimed to systematically review digital interventions to increase vegetable intake in adults to: 1) describe the effectiveness of the interventions in terms of increased consumption; 2) examine links between effectiveness and use of co-design, personalisation, behavioural theories, and/or a policy framework; and 3) identify other features that contribute to effectiveness.

## Methods

The protocol for this systematic review is registered with the international prospective register of systematic reviews (PROSPERO; CRD42022290926). The design and reporting of this review were guided by the Preferred Reporting Items for Systematic Reviews and Meta-analyses (PRISMA) statement (Additional file 1) and the synthesis without meta-analysis (SWiM) in systematic reviews reporting guidelines [29].

### Eligibility criteria

The population, intervention, comparison, outcome (PICO) framework was used to develop the inclusion and exclusion criteria for study selection. Study designs included RCT, pseudo-RCTs, and pre-post interventions. The population included community-dwelling adults (18 years and older). Studies were excluded if they included pregnant and/or lactating women and/or institutionalised adults. Studies on populations for primary and secondary prevention were included. Interventions were included if they were a digital intervention targeting knowledge of, attitudes towards, and skills in using vegetables. In this review, “digital interventions” were interventions that included any of the following digital components: applications (apps; native, web, progressive and hybrid), websites, computer programs, mobile games, Short Message Services (SMS), Social Networking Services (SNS) and wearable devices [10]. Multi-modal interventions with non-digital components (e.g., face-to-face consultations) were included if digital features represented the primary focus of the intervention. The focus of this review was on vegetable intake, so the primary outcome was change in vegetable intake (i.e., measured as serves, portions, or grams/day). Secondary outcomes considered included changes in attitudes, knowledge, skills, self-efficacy, access and/or intentions related to vegetable intake. Studies were excluded if vegetable intake could not be examined separately. Only peer-reviewed original research articles published in English were included.

### Search strategy

The search was developed in consultation with a librarian and undertaken in November 2021 and updated in August 2022. Published literature from January 2000 to August 2022 was searched. The year 2000 was selected as this coincided with an increase in the use of digital technologies in nutrition research and is in alignment with similar reviews of digital interventions [30]. The following databases were searched: MEDLINE (Complete), Embase, PsycINFO, Scopus (only extra searching), CINAHL (EbscoHost), Cochrane Library (Wiley), Rural and Remote Health database (INFORMIT), Health and society database (INFORMIT), IEEE Xplore, ClinicalTrials.gov and the Australian New Zealand Clinical Trial Registry. The full search strategy can be found in Additional file 2. Briefly, search terms were combined using the AND/OR operators for digital (‘digital, ‘smartphone’, ‘website’, ‘app’), intervention (‘intervention’, ‘randomized controlled trial’) and outcomes (‘vegetables’). Reference lists from systematic reviews identified in the search and included records were hand-searched to identify any additional studies. Where relevant protocol papers were identified during the search, an attempt was made to find the accompanying trial papers.

### Data extraction

Studies were screened using Covidence software by two members of the team (KML, LA), first by title and abstract and then by full text. Discrepancies were resolved by discussion. Duplicates were removed in Covidence. Data were extracted by one reviewer (KML) and checked by a second reviewer (LA). A data extraction template was developed and piloted in Excel specifically for this review. The following information was extracted from each study: study design (setting, intervention and control conditions, duration), intervention features (digital tools used, co-design methods, behaviour change framework and taxonomies used, personalisation, NOURISHING framework policy domains and areas), population (country, age, sex, rurality, primary or secondary prevention); outcome measures (primary or secondary outcome, change in intake, behaviour, attitude, knowledge, skills, self-efficacy, intention and/or access); results for vegetable intake and effectiveness (yes/no determined based on statistically significant results for vegetable intake).

### Data synthesis

A descriptive synthesis of the findings from the included studies was conducted. No meta-analysis was undertaken due to the heterogeneous nature of the digital tools used, characteristics of the populations in the included studies and the indicator of vegetable intake reported. The effectiveness and features of all interventions were summarised to better understand the characteristics that may increase likeliness of effectiveness. Features investigated included the population and study design, such as age, sex, rurality, use of co-design practices, behaviour change theory and personalisation methods. Studies were also mapped against the World Cancer Research Fund International’s NOURISHING framework [28]. This framework comprises three broad domains of policy actions (food environment, food system and behaviour change communication), 10 key policy areas within these domains, and the specific policy actions, which should be identified and implemented by policymakers to fit their national contexts and populations [28]. Examples of policy areas for these three domains included using economic tools to address food affordability (food environment domain), supply chain actions (food systems domain) and nutrition education and skills (behaviour change communication domain). We mapped whether the three broad domains and underlying 10 key policy areas were employed in the design of the intervention.

### Risk of bias assessment

Two authors (KML, SP) performed an independent assessment of the risk of bias on the included studies, with any discrepancies resolved by consensus. Three Cochrane Risk of Bias tools were used: for randomized trials (RoB 2), for cluster RCTs (CRCT; RoB 2 CRCT) and for non-randomized studies of interventions (ROBINS-I) [31, 32]. The RoB 2 and RoB 2 CRCT domains for risk of bias assessment included randomization process, deviations from the intended interventions, missing outcome data, measurement of the outcome and selection of the reported result. The judgement within each domain was assessed to carry forward to an overall risk of bias judgement as low risk, some concerns or high risk of bias. The ROBINS-I domains for risk of bias assessment include confounding, selection of participants, classifications of interventions, deviations from intended interventions, missing data, measurements of outcomes and selection of reported results. The judgement within each domain was used to inform an overall risk of bias judgement as either low-risk, moderate-risk, serious risk, critical risk or no information reported.

## Results

**Fig. 1:**
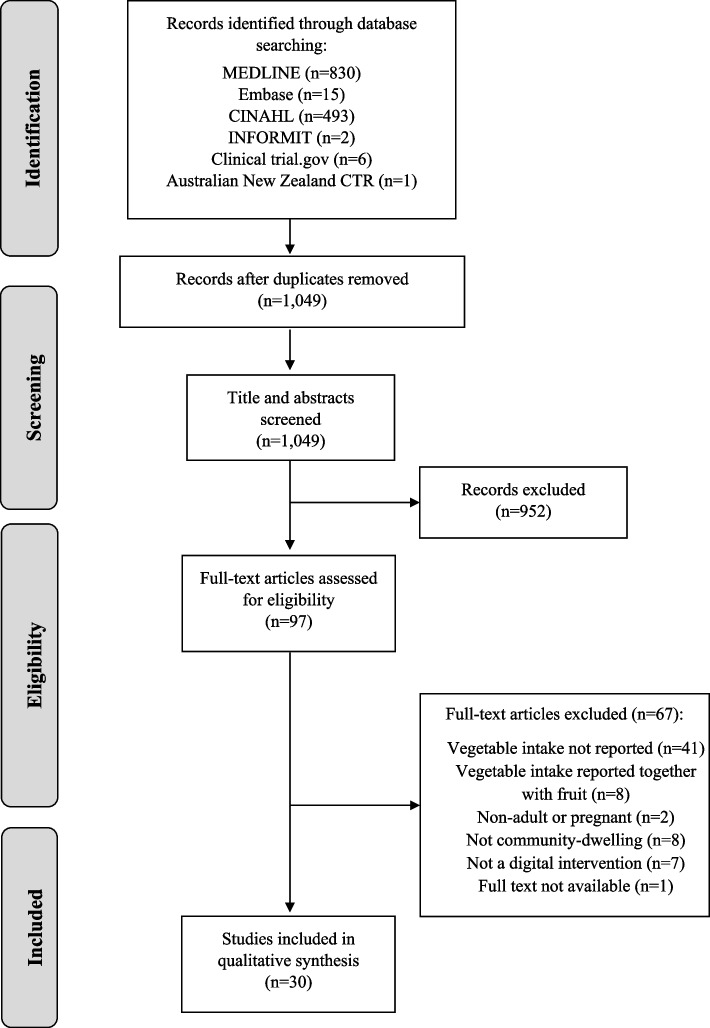
PRISMA flow diagram of study selection

The search strategy retrieved 1,347 records (Fig. 1). After the removal of duplicates, 1,049 articles were screened for inclusion based on their title and abstract. Of these, the full texts of 97 articles were screened. This review included 30 studies [33–62] (Table 1).

**Table 1:** Characteristics of included studies (*n* = 30)

Author and date	Population				Study			Outcome		Intervention results	
	**Country**	**n**	**Mean age, sex, rurality**	**Primary vs secondary prevention**	**Design**	**Duration**	**Follow-up**	**Primary**	**Secondary**	**Vegetable intake**	**Effective**
Abu-Saad 2019 [33]	Israel	50	53 y 58% female Not rural	Secondary – participants with T2DM	Pilot two-arm un-blinded RCT	6 mo	BL, 3 mo, 6 mo, 12 mo	Diabetes-related dietary knowledge	Vegetable, fruit, wholegrain, added sugars, dietary fibre intake PA, adiposity, HbA1c	NS increase vs CG (4.2 vs 3.4 portions/d)	No
Alonso-Dominguez 2019 [34]	Spain	204	61 y 46% female Not rural	Secondary – participants with T2DM	Two-arm RCT	12 mo	BL, 3 mo, 12 mo	Mediterranean Diet Adherence Screener (including ≥ 2 serves/d vegetables)	Diet Quality Index, clinical measures	NS increase adherence at 12 mo (11%) vs BL	No
Bhurosy 2020 [35]	US	165	19 y 86% female Not rural	Primary – diet	RCT	3 d	Day 1, day 2, day 3	Red/orange vegetable intake		S increase from 0.9 ± 0.9 times/d on day 1 to 1.6 ± 1.3 times/d on day 2 and to 1.3 ± 1.3 times/d on day 3. NS increase in the CG	Yes
Bozorgi 2021 [36]	Iran	120	52 y 40% female Not rural	Secondary – participants with hypertension	Two-arm RCT	6 mo	BL, 2 mo, 6 mo	Adherence to medication	DASH (including vegetable intake), blood pressure, PA	Increase in IG (*n* = 19 by > 2 serves/d; no statistical comparison)	No
Brown 2014 [37]	US	150	22 y Sex NA Not rural	Primary – diet	Pilot two-arm RCT	7 wk	BL, 7 wk	MyPlate food group recognition	Vegetable, fruit intake	Trend in increase in intervention group (data not shown)	No
Cantisano 2022 [56]	Spain	16	21 y 100% female Not rural	Primary – diet and lifestyle	Pre-post trial	3 mo	BL, 3 mo	Dietary intake (Global Diet Quality Index, 8 FG including vegetables)	PA, lifestyle and wellbeing	S increase of 3.75 score vs BL (*P* = 0.005)	Yes
Celis-Morales 2016 [57]	Ireland, NL, Spain, Greece, UK, Poland, Germany	1269	40 y 59% female Not rural	Primary – diet and PA	Four-arm RCT	6 mo	Low intensity: BL, 3, 6 mo High intensity: BL, 1, 2, 3, 6 mo	Dietary intake (9 FG including vegetables), Healthy Eating Index	Anthropometric measures (Weight, BMI, waist), biomarkers	NS increase of 2.0 g/day (*P* = 0.81) between CG and IG	No
Chan 2020 [38]	US	160	70 y 100% men Not rural	Secondary – participants with prostate cancer	Pilot four-arm RCT	3 mo	BL, 3, 6 mo	Feasibility	Diet score, dietary intake (7 FG including cruciferous vegetables), PA	S increase in vs CG (0.29 serves/d)	Yes
Debon 2020 [39]	Brazil	39	59 y 82% female Not rural	Secondary – participants with hypertension	Pilot non-blinded non- randomized, controlled trial	3 mo	BL, 3 mo	Dietary intake (10 FG including vegetables), self-care, biomarkers, blood pressure		NS increase in vegetable intake in the IG vs BL (0.95 serves/wk)	No
Elbert 2016 [40]	NL	146	41 y 73.3% female Not rural	Primary—diet	RCT	6 mo	BL, 6 mo	Fruit and vegetable intake overall and by health literacy	Self-efficacy in eating fruit and vegetables	NS increase IG vs BL. S increase in participants with high health literacy vs low	No
Fjeldsoe 2019 [41]	Australia	114	54 y 67% female Not rural	Primary—lifestyle	RCT	12 mo	BL, 6, 12 mo	Fruit, vegetable, SSB intake, takeaway meals, fat, fibre index, weight, PA		NS increase in serves/day vs BL (0.10; 95% CI: − 0.32 to 0.53)	No
Gilson 2017 [42]	Australia	19	48 y Not rural	Primary—diet and physical activity	Pilot non-randomised uncontrolled trial	5 mo	BL, 5 mo, 2 mo follow up	Fruit, vegetable, saturated fat, SSB, PA	Sedentary periods	S increase by 1 serve/d vs BL (*P* = 0.024)	Yes
Goni 2020 [43]	Spain	720	60 y 24% female Not rural	Secondary – participants with atrial fibrillation	Single-blind RCT	2 y	BL, 1, 2 y	Mediterranean diet (including vegetables)		NS increase vs CG (-20 g/day 2-y change)	No
Hansel 2017 [44]	France	120	57 y 67% females Not rural	Secondary – participants with T2DM and abdominal obesity	Two-arm open-label RCT	4 mo	BL, 3, 6 mo	International Diet Quality Index (including vegetables)	Weight, HbA1c, measured maximum oxygen consumption	S increase 0.3 points vs CG (-0.3; *P* = 0.01)	Yes
Hebden 2014 [45]	Australia	51	23 y 81% female Not rural	Secondary – participants with overweight or obesity	Pilot two-arm RCT	3 mo	BL, 3 mo	Weight, BMI	Vegetable, SSB intake, takeaway meals, PA	NS increase vs CG	No
Hendrie 2020 [58]	Australia	1224	48 y 84% female Not rural	Primary—diet	Pre-post trial	90 d	BL, 21, 90 d	Vegetable intake and variety	Psychological variables (attitudes, intentions, self-efficacy, and action planning) and app usage	S increase of 0.48 serves/d and 0.35 types /d vs BL	Yes
Jahan 2020 [46]	Bangla-desh	412	47 y 86% female Rural	Secondary – participants with hypertension	Two-arm open-label RCT	5 mo	BL, 5, 12 mo	Salt, fruit, vegetable intake, blood pressure, weight, PA	Dietary salt excretion, glucose, quality of life	NS increase (1% more increased vs CG)	No
Kerr 2016 [52]	Australia	247	24 y 65% female Not rural	Primary—diet	Three-arm RCT	6 mo	BL, 6 mo	Fruit, vegetables, SSB, energy-dense nutrient-poor foods and beverages	Weight, BMI	NS decline vs CG (-0.1 serves/d)	No
Lara 2016 [47]	UK	70	61 y 75% female Not rural	Primary—lifestyle	Pilot two-arm single-blinded RCT	2 mo	BL, 2 mo	Mediterranean diet (including vegetables), PA, healthy ageing		Decline (2.6 portions/d) vs BL (2.4 portions/d) (no statistical comparison)	No
Lombard 2016 [48]	Australia	649	40 y 100% female Rural	Primary—weight management	Cluster RCT (by town)	12 mo	BL, 12 mo	Weight loss	Diet quality, greater self-management behaviours (including vegetables)	NS increase in IG by 3 g/d	No
Perez-Junkura 2022 [59]	Spain	27	37 y 81% female Not rural	Primary—diet	Non-randomised, uncontrolled trial	12 mo	BL, 12 mo	Dietary intake (including vegetables)	Gastrointestinal symptoms	NS increase vs BL by 0.7 portions/d	No
Plaete 2015 [60]	Belgium	426	32 y 60% female Not rural	Primary—diet	Three arm- non-randomised controlled trial	1 mo	BL, 1 wk, 1 mo	Fruit, vegetable intake		S increase vs BL (IG1: χ2 1 = 5.3, *p* = 0.02; IG2: χ2 1 = 12.8, *p* < 0.001). NS increase in CG	Yes
Pope 2019 [49]	US	38	22 y 74% female Not rural	Primary—lifestyle	Two-arm, RCT	3 mo	BL, 1.5 mo, 3 mo	Feasibility	Fruit, vegetable, wholegrains, SSB, calories, PA, physiology, weight	Decline vs BL (no statistical comparison)	No
Recio-Redruguez 2016 [50]	Spain	833	52 y 62% females Not rural	Primary—diet	Two-arm RCT	3 mo	BL, 3 mo	Mediterranean diet (including vegetables), PA	Blood pressure, BMI, biomarkers	NS decline vs CG (-4% ≥ 2 serves/d)	No
Schulz 2014 [51]	NL	5055	44 y 47% female Not rural	Primary -lifestyle	Three-arm RCT	2 y	BL, 1, 2 y	Overall risk score	Fruit, vegetable intake, alcohol, smoking, PA	NS increase vs CG (β 0.07, *P* = 0.62)	No
Turner-McGrievy 2013 [53]	US	96	43 y 75% female Not rural	Secondary – participants who are overweight	Post hoc analysis of RCT	6 mo	BL, 3, 6 mo	Weight	Fruit, vegetables intake, PA	NS increase between app, paper journal or website *P* = 0.67)	No
Wang 2021 [54]	China	110	18 y 59% female Not rural	Primary—lifestyle	Non-randomized controlled trial	21 d	BL, 21 d	Dietary intake (including vegetables)	PA, fitness, body composition	S increase vs BL (0% vs 7% ≥ 500 g/d). NS increase in CG	Yes
Wang 2020 [55]	Mongolia	171	51 y 57% males Not rural	Secondary – participants with T2DM	Two-arm RCT	12 mo	BL, 12 mo	Plasma glucose	Fruit, vegetable intake, PA, smoking, weight control	S increase in % who increased intake vs CG (87% vs 29%; p < 0.001)	Yes
Williams 2022 [61]	Australia	477	52 y 78% female Not rural	Primary – diet and lifestyle	Two-arm RCT	3 mo	BL, 1, 3 mo	Healthcare professional visitations	PA, BMI, fruit, vegetable intake	NS increase in meeting guidelines vs CG (0.90 [0.39, 2.10])	No
Zenun Frano 2022 [62]	UK	187	43 y 84% female Not rural	Primary—diet	Two-arm, single-blinded RCT	3 mo	BL, 3 mo	m-AHEI (including vegetable scores)	Weight, BMI, PA	NS decline vs CG (-0.32 m-AHEI points)	No

*Abbreviations:BL* baseline, *BMI* body mass index, *CG* control group, *d*. day, *DASH* Dietary Approaches to Stop Hypertension, *FG* food groups, *IG* intervention group, *mo* month, *m-AHEI* modified-alternative healthy eating index, *NA* not available, *NS* non-significant, *PA* physical activity, *RCT* randomized controlled trial, *S* significant, *SSB* sugar-sweetened beverages, *T2DM* type 2 diabetes mellitus, *wk* week, *y* year

### Study characteristics

The 30 included studies comprised of RCTs (*n* = 22) [33–38, 40, 41, 43–47, 49–53, 55, 57, 61, 62], a CRCT (*n* = 1) [48] and non-randomized trials (*n* = 7) [39, 42, 54, 56, 58–60]. Intervention duration ranged from 3 days [35] to 2 years [43, 51]; more than half (*n* = 17; 57%) of studies had a follow-up period less than 6 months. Most studies were conducted in Australia [41, 42, 45, 48, 52, 58, 61], followed by the United States [35, 37, 38, 49, 53], Spain [34, 43, 50, 56, 59], the Netherlands [40, 51], the United Kingdom [47, 62], Belgium [60], France [44], pan-European [57], Israel [33], Iran [36], Brazil [39], Bangladesh [46], China [54] and Mongolia [55]. The studies included sample sizes ranging from 16 [56] to 5,055 [51], with 16 studies (53%) including a sample of 150 or more participants. The mean age of participants ranged from 18 years [54] to 70 years [38], with many (*n* = 19) conducted in mid-aged and older adult populations (> 40 years). Two studies delivered the digital interventions exclusively in rural areas [46, 48]. Eleven (37%) interventions recruited populations with health conditions, including hypertension [36, 39, 46], type 2 diabetes mellitus [33, 34, 44, 55], heart disease [43] prostate cancer [38] and overweight or obesity [45, 53]. The remaining studies were conducted in generally healthy populations and were designed to improve diet and/or lifestyle (*n* = 18) or weight management (*n* = 1). Over half of the studies (*n* = 17) were published since 2019.

### Risk of bias

Risk of bias within 25 (83%) studies was high or serious because of missing outcome data for RCTs or bias due to confounding in non-RCTs (Additional file 3). Most RCTs (*n* = 17) and the CRCT adequately generated and concealed allocation resulting in no imbalances apparent between groups. Participant blinding was not possible because of the nature of digital health interventions and was not considered to increase risk of bias. The measure of assessment of vegetable intake was considered appropriate in most RCTs and the CRCT except for three studies where insufficient information was provided. Assessors were blinded to the intervention received by participants in 11 studies. Assessment of the outcome could have been influenced by knowledge of intervention received. However, this was deemed unlikely due to the dietary assessment methods and protocols used to assess vegetable intake, where it is unlikely that dietary coders were aware of the intervention allocation. Finally, seven studies did not reference a protocol or trial registration with a pre-specified analysis plan that was finalized before unblinded outcome data were available for analysis, which may be due to publication preceding the development of reporting guidelines.

### Characteristics of digital tools

The most common digital tools used in the included studies were apps (*n* = 19; 63%), followed by SMS messaging (*n* = 10; 33%) and websites (*n* = 9; 30%). Some studies also used phone coaching and emails, and some interventions included a ‘dashboard’ feature to summarise resources and goals [39, 47]. Just under half (*n* = 13; 43%) used a combination of digital tools (Table 2).

**Table 2:** Summary of the features of digital interventions grouped according to effectiveness

Author and year	Control	Intervention features					
		**Intervention**	**NOURISHING policy domain and policy area**	**Digital tool**	**Co-design**^**a**^	**Behaviour change theory**	**Personalisation**
**Effective**							
Bhurosy 2020 [35]	Self-monitoring of diet	Self-monitoring of diet, including red/orange vegetable intake, set a goal to eat 1 more, take pictures of serves	Domain: BCC Policy: nutrition education and skills	App		Assigned goal setting	
Cantisano 2022 [56]		ePSICONUT programme – eHealth tools (Headspace, Insight Timer, Fabulous, YouTube channel, WhatsApp, e-mail, and Excel sheets to perform tasks/activities)	Domain: BCC Policy: nutrition education and skills	App, SMS messages, videos, email, Excel	Not true co-design: researchers and health professionals	Goal setting	
Chan 2020 [38]	Generalised dietary and PA advice	TrueNTH Community of Wellness—educational material, links to resources, self-monitoring diet and PA	Domain: BCC Policy: nutrition education and skills; nutrition advice and counselling	Website, Fitbit, SMS messages, phone calls		Social cognitive theory, goal setting	Diet and PA advice, videos and reports, exercise trainer and dietitian, dashboard
Gilson 2017 [42]		Jawbone UP™—financial incentives program. Education materials and self-monitoring PA and healthy dietary choices, and financial incentives for changing behaviours	Domains: BCC; food environment Policies: nutrition education and skills; economic tools to address affordability and purchase incentives	App, activity tracker		Goal setting	Support and feedback from researchers on goals
Hansel 2017 [44]	Generalised dietary advice	ANODE—dietetic tool providing menus, shopping list, recipes, PA prescribed	Domain: BCC Policy: nutrition education and skills; nutrition advice and counselling	Website			Menus, shopping list based on preferences, tastes, calories, needs
Hendrie 2020 [58]		VegEze – motivation and education to increase intake and variety, self-monitoring, gamification, > 50 recipes and meal suggestions	Domain: BCC Policy: nutrition education and skills	App	Not true co-design: dietitians, researchers, product developers, software engineers, adults (25–45 y)	Behaviour change wheel; motivation, goal setting, self-monitoring, social comparison, gamification	Feedback and motivational messages for meeting goals
Plaete 2015 [60]	Generalised information	MyPlan 1.0—motivation and education to improve behaviour (group 1 and 2 were recruited by GPs and researchers respectively)	Domain: BCC Policy: nutrition education and skills	Website	Not true co-design: researchers, general practitioners	Self-regulation, health action process, goal setting	Feedback on health behaviours, action plan
Wang 2021 [54]	Health education	WeChat—health education, self-monitoring, reminders, diet, sport advice and supervision	Domain: BCC Policy: nutrition education and skills	App		Trans-theoretical Model	Dietitians, sports coach advice, health reports
Wang 2020 [55]	Text messages on general health information	Text messages covering health awareness, diet control, PA, living habits, weight control	Domain: BCC Policy: nutrition education and skills; nutrition advice and counselling	SMS messages	Not true co-design: endocrinology, chronic disease, health education, disease prevention experts	Trans-theoretical Model	
**Ineffective**							
Abu-Saad 2019 [33]	Standard lifestyle counselling	Interactive Lifestyle Assessment, Counselling, and Education (I-ACE)—self-monitoring of dietary intake and PA, dietitian-delivered lifestyle education and advice	Domain: Behaviour Change Communication (BCC) Policy: nutrition education and skills; nutrition advice and counselling	App	Not true co-design: adults, dietitians	Motivational interviewing, goal setting	Clinical counselling to improve diet based on diet, ethnicity, culture, age, health status
Alonso-Dominguez 2019 [34]	Generalised dietary and PA advice	EVIDENT II—self-monitoring diet and PA, in-person food/cooking workshops, walks	Domain: BCC Policy: nutrition education and skills; nutrition advice and counselling	App	Not true co-design: software engineers, dietitians, PA experts		Diet and PA advice based on diet, PA, age, sex, weight, height, stride
Bozorgi 2021 [36]	Usual care	Education and support information on disease management, healthy diet (DASH and low-salt diet), weight loss and motivational messages	Domain: BCC Policy: nutrition education and skills; nutrition advice and counselling	App			Messages based on patient characteristics
Brown 2014 [37]	Brochure containing same information	Mobile MyPlate—behavior-directed motivational text messages on the US Dietary Guidelines and messages of the My-Plate icon	Domain: BCC Policy: nutrition education and skills	App, SMS messaging	Not true co-design: nutrition and health societies, industry, department of agriculture and health and human services	Goal setting	
Celis-Morales 2016 [57]	Generalised dietary advice	Food4Me—self-monitoring of diet and PA, three levels of feedback report: Level 1: based on diet data; Level 2: based on diet and phenotype data; Level 2: based on diet, phenotype and genotype data	Domain: BCC Policy: nutrition education and skills; nutrition advice and counselling	Website, internet forum, accelerometer		Behaviour change wheel; motivation, self-monitoring, assigned goal setting	Diet advice based on diet, phenotype (anthropometric; blood biomarkers) and/or genotype (5 nutrient-responsive genes)
Debon 2020 [39]	Health education workshops	Health education workshops. Self-monitoring of physical measurements, (e.g. blood pressure, anthropometrics, sleep, mood, PA). Recommendations based on reference values. Alerts and reminders	Domain: BCC Policy: nutrition education and skills; nutrition advice and counselling	App		Goal setting	Advice based on reference values, dashboard summary of health conditions
Elbert 2016 [40]	No health information	Text- or audio-based tailored health information, recipes, testimonials	Domain: BCC Policy: nutrition education and skills	App		Social cognitive theory, goal setting	Action plan, testimonial matching, advice based on current diet, barriers to fruit and vegetable intake and health
Fjeldsoe 2019 [41]	Brief written feedback	Get Healthy, Stay Healthy (GHSH)—extended contact intervention with text messages and phone calls with coach	Domain: BCC Policy: nutrition education and skills	SMS messages		Goal setting	Diet and PA goals, frequency of goals and texts, phone coaching
Goni 2020 [43]	Usual clinical care	PREDIMAR—nutrition education on the Mediterranean diet, self-monitoring of diet, recipes	Domain: BCC Policy: nutrition education and skills; nutrition advice and counselling	Website, app, printed resources, phone calls, cooking videos, testimonials	Not true co-design: dietitians, nutritionists, epidemiologists, doctors, chefs, programmers		Dietary advice by a dietitian
Hebden 2014 [45]	Booklet from dietitian	Booklet, text messages, emails, app and forums, recipes, self-monitoring diet and PA	Domain: BCC Policy: nutrition education and skills	SMS messages, e-mails, app, Internet forums		Transtheoretical model	Motivational advice and instantaneous diet and PA feedback based on guidelines, dietitian access
Jahan 2020 [46]	Brochure on health education	Health education on DASH, PA, generalised text messages on recommendations	Domain: BCC Policy: nutrition education and skills; nutrition advice and counselling	SMS messages			
Kerr 2016 [52]	Self-monitoring without feedback	Connecting Health and Technology (CHAT)—text messages, self-monitoring and feedback on diet using images, web links and recipes	Domain: BCC Policy: nutrition education and skills	SMS messaging, app	Not true co-design: young adults (18–30 y)	Self-determination	Feedback based on diet, name
Lara 2016 [47]	Usual care	Living, Eating, Activity and Planning through retirement (LEAP)—information on healthy eating (Mediterranean diet), recipes, PA, social roles, self-monitoring	Domain: BCC Policy: nutrition education and skills	Website	True co-design: researchers, adults (> 55 y), health social care professionals	Health action process, goal setting	Content based on demographics, diet and goals, dashboard summary
Lombard 2016 [48]	Generalised health session	HeLP-her—self-management education manual, group session, phone coaching, text messages	Domain: BCC Policy: nutrition education and skills	SMS messages, phone coaching		Self-determination, cognitive behavioural, motivational interviewing, goal setting	Diet and PA goals and action plan, by name, coaching
Perez-Junkura 2022 [59]		GlutenFreeDiet platform—dietary evaluation, which allows dietitians to measure energy content and nutrient distribution	Domain: BCC Policy: nutrition education and skills; nutrition advice and counselling	Website, app counselling			Feedback based on diet
Pope 2019 [49]	Identical, Facebook	Facebook group, self-monitoring diet and PA with smartwatch	Domain: BCC Policy: nutrition education and skills	App, smartwatch		Social cognitive, self-determination	
Recio-Redruguez 2016 [50]	Generalised information	Diet and PA counselling, leaflet, self-monitoring diet and PA	Domain: BCC Policy: nutrition education and skills	App, counselling	Not true co-design: dietitians, PA experts, software engineers		Feedback and plan based on diet and PA
Schulz 2014 [51]	Health risk appraisal	MyHealthyBehavior—health risk appraisal, feedback to improve diet, alcohol, PA, smoking (sequentially or simultaneously)	Domain: BCC Policy: nutrition education and skills	Website			Feedback based on diet, PA, smoking, by name
Turner-McGrievy 2013 [53]	Podcast	Podcast, self-monitoring diet and PA, social support	Domain: BCC Policy: nutrition education and skills	Podcast, app		Social cognitive theory, goal setting	
Williams 2022 [61]	Static text-based messages and letter from GP	Diabetes Online Risk Assessment (DORA) study—video-based story (80–144 s in duration), links to reputable healthy lifestyle resources (e.g. Nutrition Australia)	Domain: BCC Policy: nutrition education and skills; nutrition advice and counselling	Website, video, SMS messages		Health belief model	Video based on individual T2DM risk factors, gender, and age
Zenun Franco 2022 [62]	Generalised dietary advice via the eNutri web app	EatWellUK study—personalised dietary advice via the eNutri web app	Domain: BCC Policy: nutrition advice and counselling	App	Not true co-design: adults (18 y and over), nutrition professionals		Dietary advice based on current diet

*Abbreviations:BL* baseline, *BMI* body mass index, *CG* control group, *d*. day, *DASH* Dietary Approaches to Stop Hypertension, *FG* food groups, *IG* intervention group, *mo* month, *m-AHEI* modified-alternative healthy eating index, *NA* not available, *NS* non-significant, *PA* physical activity, *RCT* randomized controlled trial, *S* significant, *SSB* sugar-sweetened beverages, *T2DM* type 2 diabetes mellitus, *wk* week, *y* year

^a^ Design and development input from stakeholders identified as true co-design only if the study used this the term “co-design”

### Vegetable intake

As shown in Table 1, vegetable intake was a primary outcome in 63% of studies (*n* = 19). Of these, some studies reported vegetable intake as a component of a Mediterranean diet score (*n* = 4), International Diet Quality Index (*n* = 1), m-Alternate Healthy Eating Index [62] or an overall diet quality index for Dominican adults [56]. Vegetable intake was assessed in most studies using brief diet questions [35, 36, 41, 42, 45, 46, 54–56, 58, 60], followed by a food frequency questionnaire [33, 37–40, 48, 51, 57, 61, 62], 24 h recall [57, 59], Mediterranean diet adherence screener [34, 43, 50], and an image-based dietary assessment tool [52].

### Co-design practices

**Fig. 2:**
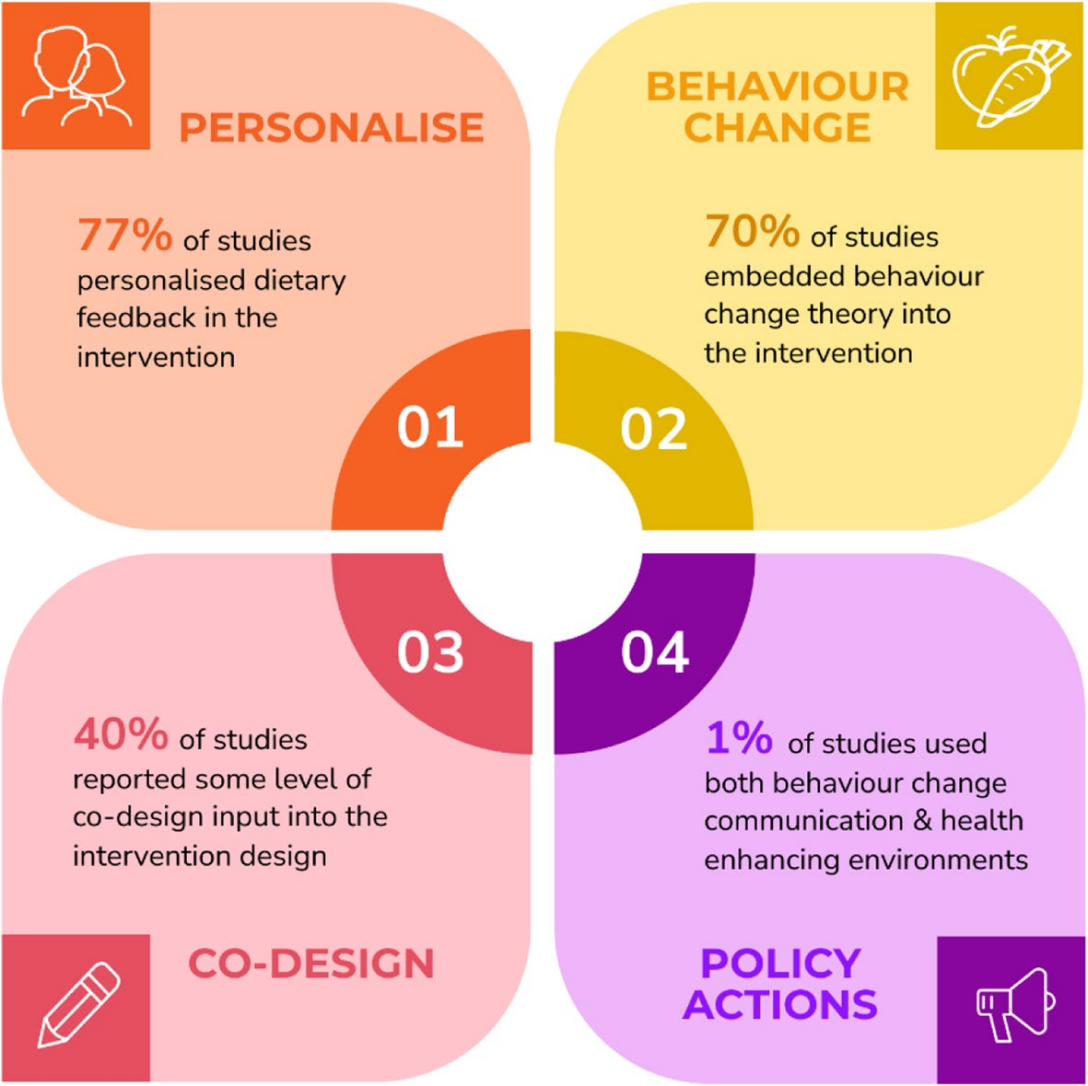
Summary of features of digital interventions to increase vegetable intake

As shown in Table 2 and Fig. 2, 40% of studies (*n* = 12) reported some level of stakeholder input into the intervention design. Only one study, by Lara et al., referred to co-design specifically; a seven-stage, sequential, iterative series of workshops were used for designing, prototyping, testing and optimising the intervention, which was undertaken with researchers, older adults (the target population) and health and social care professionals [47]. This study was designated as using true co-design. Of the studies that reported stakeholder input, health care professionals, such as dietitians and general practitioners, were the most commonly reported stakeholders involved in the design, followed by software engineers. Only five studies reported involving consumers with lived experiences, including young adults (aged 18–30 years) in the Connecting Health and Technology (CHAT) study [52], adults aged over 55 years in the Living, Eating, Activity and Planning through retirement (LEAP) study [47] and Arab adults in a trial of ethnic minority adults with type 2 diabetes mellitis [33].

### Personalisation methods

Twenty-three studies (77%) included some level of personalised intervention feedback (Table 2 and Fig. 2). The degrees of personalisation ranged from low (e.g., feedback based on assessment of current diet [52]), to moderate (e.g., personalisation of menus and shopping lists [44]), to high (e.g., individual coaching from a dietitian [38]); only one study reported offering participants the opportunity to customise their personalisation, based on preferred frequency and timing of text messaging [41]. Seven studies provided access to diet or physical activity coaching by a health professional via an app [33, 38, 41, 43, 45, 48, 54], phone calls [38, 41, 48], video calls [43], and SMS messages, emails and online forums [45]. One study personalised content to specifically address barriers to vegetable intake based on participant responses [40], another study used a digital program to design a personalised daily or weekly menu based on user preferences such as taste in foods, season and price range [44], while another study created a personalised video to promote healthy lifestyle behaviours based on age, gender and individual type 2 diabetes risk factors [61]. SMS-based interventions often used the participants’ name within the content [34, 36, 39]. Four studies provided personalised feedback and/or action plans based on demographic characteristics (such as age, sex, ethnicity and culture) and/or participant preferences [34, 36, 39] although limited information was provided on how this personalisation was designed or delivered, or whether personalisation was applied to the dietary component of the intervention. Other studies included some aspects of individualised support, although access to advice and support from dietitians was not provided [42, 62].

### Theoretical underpinning and framework

Twenty-one studies (70%) reported embedding behaviour change theories into intervention design and delivery. Social cognitive theory and the trans-theoretical model were the two theories/models used most to underpin the interventions, with behaviour change techniques such as goal setting, motivational interviewing or action planning most frequently used (Table 2 and Fig. 2). When mapping against the NOURISHING framework, all studies aligned with the behaviour change communication domain, with the two policy areas of “nutrition education and skills”, and “nutrition advice and counselling in health care settings” identified. One study also mapped to the food environment domain, with the policy area of “economic tools to address affordability and purchase incentives” identified [42]. In this study, participants accumulated points and received a monetary reward at the end of the intervention relative to the number of healthy dietary choices logged. No studies aligned with the food system domain.

### Effectiveness of digital interventions

Only nine studies (30%) reported statistically significant improvements in vegetable intake (i.e., designated as effective interventions) compared with a control group [38, 44, 55] or compared with baseline. In the latter case, this included pre-post interventions [56, 58], uncontrolled randomised trials [42] and RCTs with no statistically significant increase in the control group (and no statistical comparison for between-group changes reported) [35, 54, 60]. There was heterogeneity in the method of reporting improvements in vegetable intake among effective studies, including serves/day and adherence to guidelines. Three studies reported change in serves/day, with the magnitude of this improvement ranging from 0.29 serves/day [38] to 1 serve/day [42]. One study reported that 87% of participants improved vegetable intake compared to 29% of the control group [55], while another study reported a 7% increase in adherence to ≥ 500 g/day of vegetables compared to baseline (and a non-significant increase in the control group) [54]. One pre-post study reporting a 3.75 points increase in vegetable score (as a component of the Global Diet Quality Index; maximum score 100) compared with baseline [56]. Two studies also reported improvements in vegetable intake, but limited data on the magnitude were provided and no statistical comparisons were reported [36, 37]. Three studies reported a decline in vegetable intake compared with baseline, including a 0.2 portion per day decline [47], a 4% decline in participants consuming ≥ 2 serves/day [50] and a further study did not report any data on the magnitude of change [49]. No studies included in this review reported on attitudes towards, knowledge of, skills in respect of, self-efficacy, access to and/or intentions with respect to vegetables.

### Features of effective digital interventions

Of the nine effective interventions, sample sizes ranged from 120 to 171 participants (Table 1). A slightly greater percentage of effective interventions were in healthy populations (*n* = 6/9; 67%) compared with the ineffective interventions (*n* = 13/21; 62%). Almost half of effective interventions were in younger adults (< 40y; *n* = 4, 44%), compared with 19% (*n* = 4) of ineffective interventions. Neither of the two interventions delivered exclusively in rural communities were effective. Vegetable intake was the primary outcome in 78% (*n* = 7) of the effective interventions, compared with 57% (*n* = 12) of the ineffective interventions.

Of the effective interventions, 33% (*n* = 3) utilised an app [35, 54, 58], 22% (*n* = 2) used a website [44, 60] and 11% (*n* = 1) used SMS messages [55] in isolation, while one study used an app and activity tracker [42] and two studies utilised a combination of four or more delivery modalities (including apps, emails, SMS messages, phone calls, videos and websites) [38, 56]. As shown in Table 2, this contrasted with the ineffective interventions, where 29% (*n* = 6) utilised an app [33, 34, 36, 39, 40, 62], 10% (*n* = 2) used a website [47, 51], and 10% (*n* = 2) used SMS messages [41, 46] in isolation, while 52% (*n* = 11) used a combination of delivery modalities [37, 43, 45, 48–50, 52, 53, 57, 59, 61].

**Fig. 3:**
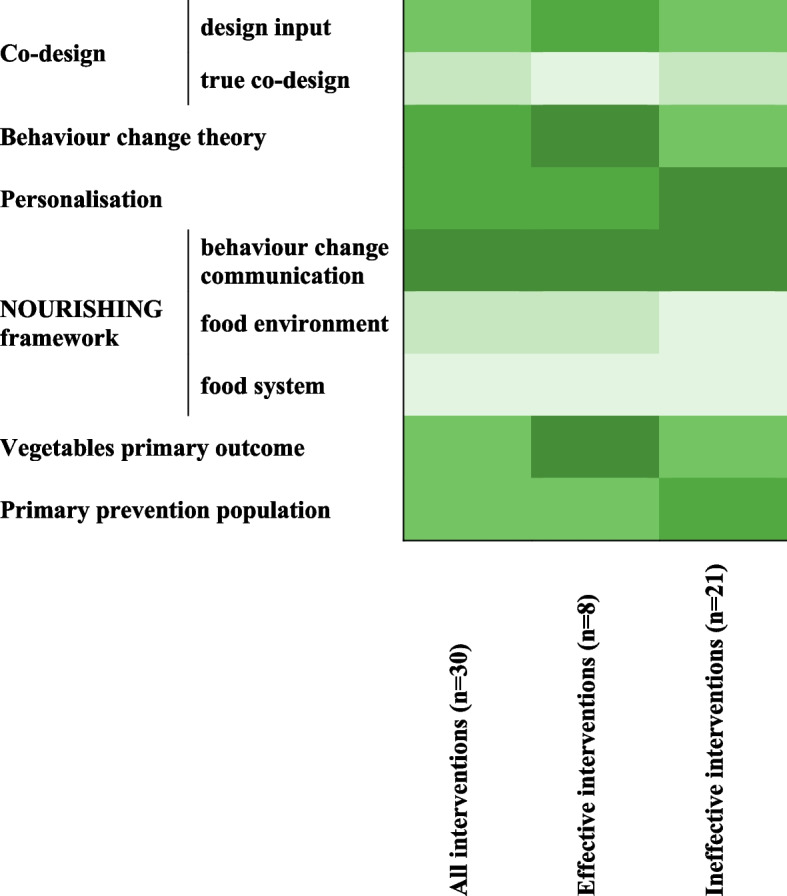
Heat map summary of features of effective and ineffective interventions to increase vegetable intake

The features of effective and ineffective interventions are compared in Fig. 3. Eighty nine percent (*n* = 8) of the effective studies referenced behavioural theories in their design (Table 2), including the trans-theoretical model theory [55], the social cognitive theory [38] and the health action process [60]. In contrast, 61% (*n* = 12) of the ineffective interventions referenced theories. Sixty-seven percent (*n* = 6) effective interventions delivered personalised information, which included personalised dietary advice from a dietitian [34, 54] and personalised menus and food shopping lists based on taste preferences and calorie needs [44]. Of the ineffective interventions, 81% (*n* = 17) included personalisation methods. Forty-four percent (*n* = 4) of the effective interventions included some level of input from stakeholders into the design of the intervention, compared with 38% (*n* = 8) of the ineffective interventions. This included design input from health care professionals, such as dietitians and general practitioners, and software engineers, but rarely involved meaningful consumer involvement. Only one (ineffective) intervention included true co-design, with iterative workshops with researchers, older adults (the target population) and health and social care professionals (Fig. 3).

## Discussion

In this systematic review we identified a paucity of digital interventions that were effective at increasing vegetable intake in adults. Embedding of behaviour change theories and inclusion of stakeholders in the design of the intervention were more common among effective interventions. We also observed that personalisation did not appear to be a feature of effective interventions. However, personalisation methods varied considerably, thus it is possible that the nature or degree of personalisation did not meet the needs of the user. Use of more comprehensive co-design methods may help to ensure that personalisation approaches are informed by the needs of the target population.

This review found that researchers used multiple, heterogenous indictors of vegetable intake when reporting outcomes from interventions, which prohibited quantitative synthesis of the magnitude of change in vegetable intake. Nevertheless, in the studies that reported serves/day, vegetable intake increased by between 0.29 to 1 serve/day, which is comparable to evidence from mass media campaigns (0.6 serves/day) [63] and workplace interventions (0.32 serves/day) [64]. Reviews of the effectiveness of interventions to increase vegetable intake specifically are lacking. Our exclusion of studies that did not report intakes of fruit and vegetables separately was critical for discerning how interventions impacted on vegetable intake alone. Given the considerable health and economic benefit at the population level of even a small increase in vegetable intake [65], future research should report these outcomes consistently, and separately from fruit intake. Further, some studies in this review reported vegetable intake as a secondary outcome, or as part of an overall diet quality scores, such as the Mediterranean diet [47, 50]. As a result, interventions targeting more than just vegetable intake may have dedicated less resources to increasing vegetable intake per se and may not have been suitably powered to detect effects on vegetable intake. Although the use of different indicators did not help explain any differences in intervention effectiveness, future interventions should report the magnitude of between-group changes in vegetable intake to ensure that results can be included in a quantitative synthesis.

Degrees of personalisation varied considerably between studies, with no clear difference in the type or level of personalisation between effective and ineffective interventions. Moreover, understanding of personalisation methods used in the included studies was limited because the reporting of the design and delivery of personalisation was often minimal. Nonetheless, while many studies used personalised feedback and/or action plans based on demographic characteristics and/or participant preferences, only one study offered participants the ability to customise the timing and delivery of their personalised content [41]. A recent study of the personalisation of digital health information identified that the preferred approach differed by age group, where young adults were more satisfied with user-driven personalisation as distinct from system-driven personalisation [66]. While system-driven personalisation offers the advantage of lower cognitive load for the user, a user-driven approach offers a greater sense of autonomy. As a result, certain population groups, such as those with higher digital health literacy, may wish to exert more control over their personalisation [67]. This degree of autonomy should be considered when designing more sophisticated approaches to personalisation, such as artificial intelligence algorithms and machine learning [68]. Digital technologies are well suited to delivering large-scale personalised dietary support, because the content, frequency and timing of the intervention can be modified to meet the needs and preferences of the user [15]. Thus, future digital interventions for increasing vegetable intake may be improved by better reporting of the use of personalisation methods, ensuring that the tool has sufficient flexibility for the content and modality to be personalised and by considering the use of more sophisticated digital techniques to achieve personalisation.

Embedded behaviour change theories were common in both the effective and ineffective interventions. There was no clear difference in the application of these theories between effective or ineffective interventions. However, it is worth noting that all interventions, bar one [42], mapped to the behaviour change communication domain of policy actions outlined in the NOURISHING framework and did not map to the food environment or food system domains. This contrasts with a recent review of settings-based and digital interventions, where studies often mapped to the food environment domain, by including strategies such as free provision of fruit and vegetables in workplaces [5]. In addition, in the review by Wolfenden et al., all interventions that mapped to the food environment domain were effective at increasing fruit and vegetable intake. The lack of behaviour change strategies at the food environment level identified in our review requires further attention in future research. For example, food prescription programs that aim to improve the accessibility and affordability of healthy foods have shown promise for improving vegetable intake and reducing food insecurity [69], and could be integrated into digital healthcare interventions via partnerships with relevant stakeholders, such as health care providers, food markets or foodbanks. This is particularly important in the era of the COVID-19 pandemic, which has increased consumer acceptance and use of digital health initiatives [70], as well as stimulated a concerted global investment in building more food secure communities [71, 72].

A paucity of studies in this review included diverse populations. Similar to other reviews of digital interventions [73], most study populations were female-skewed, and of mid or older age (> 40 years). Disadvantaged populations, such as those with lower socio-economic position and who are culturally and linguistically diverse, were under-represented. Thus, there is potential for selection bias and response bias to have limited the generalisability of the findings from these studies. In addition, the “digital divide” persists, where lower income countries, racial/ethnic minorities, older adults, and individuals who live in lower income households and rural areas have less access to the internet and lower digital literacy [74]. However, global internet use has doubled from 33 to 65% in the last decade [16], and there is some evidence that digital inclusion is increasing [10, 11, 75]. Therefore, there is an opportunity to test the effectiveness of digital interventions in diverse populations to help reduce dietary (and health) inequities and improve digital literacy. Moreover, findings from this review confirm recent research highlighting a lack of nutrition research in rural settings, where there is inequitable access to healthcare and fresh produce, such as fruit and vegetables [13]. As a result, future interventions should consider external validity in other less well-represented population groups such as individuals with lower socioeconomic position and those living in rural settings. Digital interventions are well suited to achieve this because of their potential for linguistic and cultural localisation, national scalability at relatively low cost, and the global drive to improve digital health equity in rural and disadvantaged communities.

Fewer than half of included studies reported on interventions that had been developed with some level of design input from stakeholders. In addition, intervention end users were very rarely involved and only one intervention specifically mentioned the use of co-design approaches. Recent reviews on the use of co-design have shown mixed findings, with one review of co-design in health settings showing widespread use [24], and another review of co-design in nutrition and health interventions in community-dwelling adults identifying no interventions implementing a complete co-design process [25]. A more recent review of the use of co-design specifically in nutrition interventions delivered within a healthcare, community or academic setting identified only two studies reporting a partnership with consumers across all stages of research [76]. Taken together, these findings reinforce the need for consistent use of co-design terminology, better reporting of design and development processes and more widespread utilisation of a translational framework for the evaluation of health interventions, such as the NASSS (non-adoption, abandonment, scale-up, spread, sustainability) framework [77]. Future research should include co-design methods at multiple levels (i.e., stakeholders with lived experience as well as technical expertise) and include stakeholders throughout, from project conception to dissemination.

Outcomes from this research have implications for the use of digital tools to improve public health nutrition and provide insights into future research needs. Despite the potential for digital tools to improve access to dietary interventions, the persistent threat that digital technologies can exacerbate social inequities of health remains [78]. As such, the inclusion of diverse populations groups in the design and implementation of digital interventions remains a priority. Without this, there is a risk that some population groups may experience barriers to the use of digital technologies, including individuals experiencing socio-economic disadvantage, individuals with disabilities, individuals who require cultural adaptations, and those with low food and digital literacy and self-efficacy [79]. Countries with diverse geographic settings and the potential for disparities in internet access, such as Australia, should ensure that digital interventions are tested in rural settings, which would otherwise be a missed opportunity for addressing widening health disparities [80]. Further, with a paucity of co-design research and consideration of environmental influences, this research suggests that the design of digital interventions to increase vegetable intake is not yet optimal in maximising effectiveness.

This review has several strengths and limitations. The main strength was the systematic approach used to search, screen, and synthesise the literature, including the PROSPERO registration of the review protocol and the use of Cochrane risk of bias tools. By limiting the search to articles published in English and including experimental study designs only, it is possible that studies that would be informative for the design of future interventions were missed. As most studies included in this review were rated as high risk for bias, findings should be interpreted with caution. Due to the heterogenous study populations and intervention designs, including small sample sizes, no quantitative synthesis could be performed. Further, intervention outcomes for vegetable intake will be subject to misreporting biases due to the self-report nature of dietary assessment tools available, which includes the potential for participants to introduce bias as their food literacy and understanding of dietary reporting improves. Lastly, grey literature and commercial products for dietary behaviour change were excluded, which may have limited our ability to capture evidence of co-design research and the full range of digital tools designed to increase vegetable intake.

## Conclusions

Few digital interventions have been effective in increasing vegetable intake among adults. Embedding behaviour change theories and involving stakeholders in intervention design may increase the likelihood of effectiveness. Personalisation was not a distinctive feature of effective digital interventions, however, this feature remains poorly understood due to considerable variation in its design and reporting. There is an unmet opportunity for the use of more comprehensive co-design methods to ensure personalisation approaches meet the needs of target populations. Furthermore, future digital interventions should consider strategies that address both behaviour change and food environment influences.

## Supplementary Information

**Additional file 1.** PRIMSA checklist.

**Additional file 2.** Search strategy.

**Additional file 3.** Cochrane Risk of Bias for RCTs, cluster RCTs and non-RCTs.

## Data Availability

Not applicable.

## Abbreviations

BMI: Body mass index
BL: Baseline
FFQ: Food frequency questionnaire
PRISMA: Preferred reporting items for systematic reviews and meta-analyses
RCT: Randomized controlled trial
RoB 2: Risk of bias in randomized trials
ROBINS-I: Risk of bias in non-randomized studies of interventions
