# Supplementary material for: Digital behaviour change interventions to increase vegetable intake in adults: a systematic review
Source: Int J Behav Nutr Phys Act. 2023 Mar 27;20:36. doi: 10.1186/s12966-023-01439-9 (PMC10042405; doi:10.1186/s12966-023-01439-9)
Supplement: Supplementary file 2 — Additional file 2. Search strategy. [file 12966_2023_1439_MOESM2_ESM.docx]

**Additional File 2**. Search strategy for MEDLINE, EMBASE, CINAHL, INFORMIT, IEEE Xplore, ClinicalTrial.gov

**MEDLINE**

| **Searches** |
| --- |
| “Mobile applications”/ or *Webcasts as topic/ or “*social networking”/ or *smartphone/ or “*text messaging”/ or “video games”/ |
| AND |
| (webcast* or podcast* or “mobile application*” or smartphone* or iphone or android or “text messag*” or app or apps or “virtual reality” or ipad* or “tablet comput*” or “tablet device*” or “instant messag*” or SMS* or whatsapp or Youtube* or “video gam*”) |
| AND |
| (e-health* or ehealth* or “electronic health*” or e-technolog* or etechnolog* or “digital health*” or “digital technolog*” or “mobile health*” or mhealth* or m-health* or e-learning* or elearning* or e-medicine* or emedicine* or e-therap* or etherap* or “health information technolog*” or e-support*) |
| AND |
| (*Telecommunications/ or *Computers/ or *computers, handheld/ or *microcomputers/ or "User-computer interface"/ or *Internet/) and (Learning/ or “Health education”/ or “Self care”/ or “Self help groups”/ or “*Teaching materials”/ or *”Health knowledge”, or attitude*, practice/ or *communication/ or *counseling/ or “*consumer health information”/ or *Therapy, “computer assisted”/ or “*health services accessibility”/ or “*delivery of health care” or “digital technology”) |
| AND |
| ((online or on-line or digital* or electronic or computer* or software or internet* or web or website* or technology-based or interactiv* or telecommunicat* or "information and communication technolog*" or ICT) (support or “self help” or chat or communicat* or “self care” or “self manag*” or “self efficac*” or selfefficac* or intervention* or education* or training or learning or teaching or “health information*” or “information service*” or lifestyle* or “life style*” or motivat* or healthcare or “health care” or complian* or adheren* or monitor* or delivery or behaviour* or behavior* or “health promotion*” or “home health*” or “home care” or counsel* or decision or tool*)) |
| AND |
| “healthy lifestyle”/ or “healthy diet”/ or Fruit/ or snacks/ or meals/ or “Health Promotion”/ or “Eating Behavior”/ or “Food Preferences”/ or Diet/ or nutrition/ |
| AND |
| Vegetable* |
| AND |
| knowledge/ or selfefficac*/ or “self efficac*”/ or skills/ or “food confidence”/ or “food involvement”/ or cook*/ or intake/ or consumption/ or attitude*/ or perception*/ or access |
| AND |
| (randomized controlled trial or controlled clinical trial or clinical study).pt. or random* adj3 trial or clinical trials as topic/ or trial.ti. or intervention/ |

**EMBASE**

| Mobile applications or Webcasts or social networking or smartphone or text messaging or video games |
| --- |
| AND |
| webcast* or podcast* or mobile application* or smartphone* or iphone or android or text messag or app or apps or virtual reality or ipad* or tablet comput* or tablet device or instant messag* or SMS or whatsapp or Youtube* or video gam* |
| AND |
| e-health* or ehealth* or electronic health* or e-technolog* or etechnolog* or digital health* or digital technolog* or mobile health* or mhealth* or m-health* or e-learning* or elearning* or e-medicine* or emedicine* or e-therap* or etherap* or health information technolog* or e-support* |
| AND |
| Telecommunications or Computers or computers handheld or microcomputers or User-computer interface or Internet or Learning or Health education or Self care or Self help groups or Teaching materials or Health knowledge or attitude practice or communication or counseling or consumer health information or Therapy computer assisted or health services accessibility or delivery of health care or digital technology |
| AND |
| online or on-line or digital or electronic or computer* or software or internet* or web or website* or technology-based or interactiv* or telecommunicat* or information and communication technolog* or ICT or support or self help or chat or communicat* or self care or self manag* or self efficac* or selfefficac* or intervention* or education* or training or learning or teaching or health information* or information service or lifestyle* or life style* or motivat* or healthcare or health care or complian* or adheren* or monitor* or delivery or behaviour* or behavior* or health promotion* or home health* or home care or counsel* or decision or tool* |
| AND |
| healthy lifestyle or healthy diet or Fruit or snacks or meals or Health Promotion or Eating Behavior or Food Preferences or Diet or nutrition |
| AND |
| Vegetable* |
| AND |
| knowledge or selfefficac* or self efficac* or skills or food confidence or food involvement or cook* or intake or consumption or attitude* or perception* or access |
| AND |
| randomized controlled trial or controlled clinical trial or clinical study |

**CINAHL**

| AB (Mobile applications or Webcasts or social networking or smartphone or ‘text messaging’ or ‘video games’) |
| --- |
| AND |
| AB (webcast* or podcast* or ‘mobile application*’ or ‘smartphone*’ or iphone or android or ‘text messag’ or app or apps or ‘virtual reality’ or ipad* or ‘tablet comput*’ or ‘tablet device’ or ‘instant messag*’ or SMS or whatsapp or Youtube* or ‘video gam*’) |
|  |
| AB (e-health* or ehealth* or ‘electronic health*’ or e-technolog* or etechnolog* or ‘digital health*’ or ‘digital technolog*’ or ‘mobile health*’ or mhealth* or m-health* or e-learning* or elearning* or e-medicine* or emedicine* or e-therap* or etherap* or ‘health information technolog*’ or e-support*) |
|  |
| AB (Telecommunications or Computers or computers handheld or microcomputers or ‘User-computer interface’ or Internet or Learning or ‘Health education’ or ‘Self care’ or ‘Self help groups’ or ‘Teaching materials’ or ‘Health knowledge’ or attitude practice or communication or counseling or ‘consumer health information’ or Therapy ‘computer assisted’ or ‘health services accessibility’ or ‘delivery of health care’ or ‘digital technology’) |
| AND |
| AB (online or on-line or digital or electronic or computer* or software or internet* or web or website* or technology-based or interactiv* or telecommunicat* or ‘information and communication technolog*’ or ICT or support or ‘self help’ or chat or communicat* or ‘self care’ or ‘self manag*’ or ‘self efficac*’ or selfefficac* or intervention* or education* or training or learning or teaching or ‘health information*’ or ‘information service’ or lifestyle* or ‘life style*’ or motivat* or healthcare or ‘health care’ or complian* or adheren* or monitor* or delivery or behaviour* or behavior* or ‘health promotion*’ or ‘home health*’ or ‘home care’ or counsel* or decision or tool* ) |
| AND |
| AB (healthy lifestyle’ or ‘healthy diet’ or Fruit or snacks or meals or ‘Health Promotion’ or ‘Eating Behavior’ or ‘Food Preferences’ or Diet or nutrition) |
| Vegetable* |
| AB (knowledge or selfefficac* or ‘self efficac*’ or skills or ‘food confidence’ or ‘food involvement’ or cook* or intake or consumption or attitude* or perception* or access) |
| AB (randomized controlled trial or controlled clinical trial or clinical study or random or clinical trials as topic or trial or intervention) |

**INFORMIT (health & society/ Rural & Remote Health)**

| Mobile applications or Webcasts or social networking or smartphone or text messaging or video games |
| --- |
| AND |
| webcast* or podcast* or mobile application* or smartphone* or iphone or android or text messag or app or apps or virtual reality or ipad* or tablet comput* or tablet device or instant messag* or SMS or whatsapp or Youtube* or video gam* |
| AND |
| e-health* or ehealth* or electronic health* or e-technolog* or etechnolog* or digital health* or digital technolog* or mobile health* or mhealth* or m-health* or e-learning* or elearning* or e-medicine* or emedicine* or e-therap* or etherap* or health information technolog* or e-support* |
| AND |
| Telecommunications or Computers or computers handheld or microcomputers or User-computer interface or Internet or Learning or Health education or Self care or Self help groups or Teaching materials or Health knowledge or attitude practice or communication or counseling or consumer health information or Therapy computer assisted or health services accessibility or delivery of health care or digital technology |
|  |
| online or on-line or digital or electronic or computer* or software or internet* or web or website* or technology-based or interactiv* or telecommunicat* or information and communication technolog* or ICT or support or self help or chat or communicat* or self care or self manag* or self efficac* or selfefficac* or intervention* or education* or training or learning or teaching or health information* or information service or lifestyle* or life style* or motivat* or healthcare or health care or complian* or adheren* or monitor* or delivery or behaviour* or behavior* or health promotion* or home health* or home care or counsel* or decision or tool* |
| AND |
| healthy lifestyle or healthy diet or Fruit or snacks or meals or Health Promotion or Eating Behavior or Food Preferences or Diet or nutrition |
| AND |
| Vegetable* |
| AND |
| knowledge or selfefficac* or self efficac* or skills or food confidence or food involvement or cook* or intake or consumption or attitude* or perception* or access |
| AND |
| randomized controlled trial or controlled clinical trial or clinical study or random or clinical trials as topic or trial or intervention |

**IEEE Xplore**

| "Full Text Only":Mobile applications” or “social networking” or smartphone or “*text messag*” or “video games” |
| --- |
| AND |
| "Full Text Only":e-health or ehealth or “electronic health” or “digital health” or “digital technolog*” or “mobile health” or mhealth or m-health or “health information technolog*” or e-support |
| AND |
| "Full Text Only":“healthy lifestyle” or “healthy diet” or fruit or snack or meal or “health promotion” or “eating behavior” or “food preference” or diet or nutrition |
| "Full Text Only":vegetable |
| "Full Text Only":knowledge or selfefficac* or “self efficac*” or skills or “food conficdene” or “food involvement” or cook or intake or consumption or attitude or perception or access |
| AND |
| "Full Text Only":randomized controlled trial or controlled clinical trial or clinical study.pt. or random* adj3 trial or clinical trials as topic or trial.ti. or intervention |

**ClinicalTrial.gov**

| Mobile or smartphone or digital or ehealth or app |
| --- |
| AND |
| knowledge or self efficacy or skills or food confidence or food involvement or cook or intake or consumption or attitude or perception or access |
