# Supplementary material for: Digital behaviour change interventions to increase vegetable intake in adults: a systematic review
Source: Int J Behav Nutr Phys Act. 2023 Mar 27;20:36. doi: 10.1186/s12966-023-01439-9 (PMC10042405; doi:10.1186/s12966-023-01439-9)
Supplement: Supplementary file 3 — Additional file 3. Cochrane Risk of Bias for RCTs, cluster RCTs and non-RCTs. [file 12966_2023_1439_MOESM3_ESM.docx]

**Additional File 3a**. Risk of Bias of included randomized controlled trials (n=19) using the revised Cochrane risk-of-bias tool for randomized trials (RoB 2)

| **Study** | | **Randomization process** | | | | **Deviations from the intended interventions** | | | | | | | | **Missing outcome data** | | | | | **Measurement of the outcome** | | | | | | **Selection of the reported result** | | | | **Overall risk-of-bias judgement** |
| --- | --- | --- | --- | --- | --- | --- | --- | --- | --- | --- | --- | --- | --- | --- | --- | --- | --- | --- | --- | --- | --- | --- | --- | --- | --- | --- | --- | --- | --- |
| **First author, Year** | **Study design** | **Allocation sequence** | **Allocation concealment** | **Baseline difference between groups** | **Risk-of-bias judgement** | **Participant blinding** | **Personnel blinding** | **Protocol deviations** | **Protocol deviations impact on outcome** | **Protocol deviations balanced between groups** | **Appropriate analysis** | **Impact of non-intention to treat analysis** | **Risk-of-bias judgement** | **Outcome data available for nearly all participants** | **Result not biased by mussing outcome data** | **Missingness dependent on true value** | **Likely that missingness dependent on true value** | **Risk-of-bias judgement** | **Outcome assessment appropriate** | **Outcome measurement differed between groups** | **Outcome assessors aware of participant intervention** | **Outcome influenced by knowledge of intervention received** | **Assessment of outcome influenced by knowledge of intervention** | **Risk-of-bias judgement** | **Follow a pre-specified analysis plan** | **Selective reporting of outcome within the outcome domain** | **Selective reporting of outcome based on multiple analyses of data** | **Risk-of-bias judgement** |  |
| **Abu-Saad 2019** | RCT* | Yes | Yes | No | **LR** | Yes | Yes | PN | N/A | N/A | Yes | N/A | **LR** | PN | No | NI | NI | **High** | No | No | No | PY | PN | **LR** | NI | No | No | **SC** | **HR** |
| **Alonso-Domínguez 2019** | RCT | Yes | Yes | No | **LR** | Yes | Yes | PN | N/A | N/A | Yes | N/A | **LR** | Yes | No | PY | PY | **LR** | No | No | No | PY | PN | **LR** | Yes | No | No | **LR** | **LR** |
| **Bhurosy 2020** | RCT | PY | NI | No | **LR** | No | No | PN | N/A | N/A | No | Yes | **HR** | No | No | PY | PY | **High** | PN | PN | No | No | No | **LR** | NI | NI | NI | **SC** | **HR** |
| **Bozorgi 2021** | RCT | Yes | Yes | No | **LR** | Yes | Yes | PN | N/A | N/A | Yes | N/A | **LR** | Yes | No | PY | PY | **LR** | No | No | PY | PY | PN | **LR** | NI | NI | NI | **SC** | **SC** |
| **Brown 2014** | RCT* | PN | NI | NI | **HR** | PY | PY | PN | N/A | N/A | PN | PY | **HR** | No | No | PY | PY | **HR** | NI | NI | PY | PY | NI | **HR** | NI | NI | NI | **SC** | **HR** |
| **Celis-Morales 2016** | RCT | Yes | NI | No | **LR** | PY | PY | PN | N/A | N/A | Yes | N/A | **LR** | No | No | PY | PY | **HR** | No | No | No | PY | PN | **LR** | Yes | No | No | **LR** | **HR** |
| **Chan 2020** | RCT* | Yes | Yes | No | **LR** | PY | NI | PN | N/A | N/A | Yes | No | **LR** | No | No | PY | PY | **HR** | No | PN | NI | PY | PN | **LR** | Yes | No | No | **LR** | **HR** |
| **Elbert 2016** | RCT | NI | PY | No | **SC** | PY | NI | PN | N/A | N/A | Yes | N/A | **LR** | No | No | PY | PY | **HR** | No | No | No | No | PN | **LR** | NI | NI | NI | **SC** | **HR** |
| **Fjeldsoe 2019** | RCT | Yes | Yes | No | **LR** | PY | No | PN | N/A | N/A | Yes | N/A | **LR** | No | No | PY | PY | **HR** | No | PN | No | PY | PN | **LR** | Yes | No | No | **LR** | **HR** |
| **Goni 2020** | RCT | Yes | Yes | Some | **LR** | Yes | No | PN | N/A | N/A | Yes | N/A | **LR** | No | No | PY | PY | **HR** | No | PN | No | PY | PN | **LR** | Yes | No | No | **LR** | **HR** |
| **Hansel 2017** | RCT | Yes | Yes | No | **LR** | Yes | No | PN | N/A | N/A | Yes | N/A | **LR** | No | No | PY | PY | **HR** | No | PN | No | PY | PN | **LR** | Yes | No | No | **LR** | **HR** |
| **Hebden 2014** | RCT* | Yes | No | No | **SC** | Yes | Yes | PN | N/A | N/A | Yes | N/A | **LR** | No | No | PY | PY | **HR** | No | PN | PY | PN | NI | **LR** | NI | NI | NI | **SC** | **HR** |
| **Jahan 2020** | RCT | Yes | Yes | No | **LR** | PY | PY | PN | N/A | N/A | Yes | N/A | **LR** | Nearly all | PY | N/A | N/A | **LR** | NI | PN | PY | PY | PN | **HR** | NI | NI | NI | **SC** | **HR** |
| **Kerr 2016** | RCT | Yes | Yes | No | **LR** | PY | PY | PN | N/A | N/A | Yes | N/A | **LR** | No | No | PY | PY | **HR** | No | PN | NI | PY | NI | **HR** | Yes | No | No | **LR** | **HR** |
| **Lara 2016** | RCT* | Yes | Yes, | No | **LR** | Yes | Yes | No | N/A | N/A | Yes | N/A | **LR** | Nearly all | No | PY | PY | **LR** | No | PN | No | PY | PN | **LR** | Yes | No | No | **LR** | **LR** |
| **Pope 2019** | RCT* | Yes | No | No | **SC** | Yes | Yes | PN | N/A | N/A | Yes | N/A | **LR** | Nearly all | No | PY | PY | **LR** | No | PN | NI | PY | PN | **LR** | Yes | No | No | **LR** | **SC** |
| **Recio-Rodriguez 2016** | RCT | Yes | Yes | No | **LR** | Yes | Yes | PN | N/A | N/A | Yes | N/A | **LR** | No | No | PY | PY | **HR** | No | PN | PY | PY | PN | **LR** | Yes | No | No | **LR** | **HR** |
| **Shulz 2014** | RCT | Yes | NI | Some | **SC** | PY | PY | PN | N/A | N/A | Yes | N/A | **LR** | No | No | PY | PY | **HR** | PN | PN | NI | PY | PY | **SC** | Yes | No | No | **LR** | **HR** |
| **Turner-McGrievy 2013** | RCT | Yes | Yes | No | **LR** | Yes | Yes | PN | N/A | N/A | Yes | N/A | **LR** | Nearly all | No | PY | PY | **LR** | No | PN | PY | PY | PN | **LR** | Yes | No | No | **LR** | **LR** |
| **Wang 2020** | RCT | PY | NI | No | **LR** | PY | PY | NI | NI | NI | No | Yes | **HR** | No | NI | PY | PY | **HR** | NI | NI | PY | PY | NI | **SC** | PY | PN | PN | **SC** | **HR** |
| **Williams 2022** | RCT | Yes | Yes | No | **LR** | No | No | PN | N/A | N/A | Yes | N/A | **LR** | No | No | PY | PY | **HR** | No | No | No | PY | PN | **LR** | Yes | No | No | **LR** | **HR** |
| **Zenun Franco 2022** | RCT | Yes | Yes | No | **LR** | No | No | PN | N/A | N/A | Yes | N/A | **LR** | No | No | PY | PY | **HR** | No | No | No | PY | PN | **LR** | Yes | No | No | **LR** | **HR** |

HR, high risk; N/A, not applicable; NI, no information; LR, low risk; PN, probably no; PY, probably yes; RCT, randomized controlled trial; SC, some concern; * indicates pilot RCT

**Additional File 3b.** Risk of Bias of included cluster randomised controlled trials (n=1) using the revised Cochrane risk-of-bias tool for cluster-randomized trials (RoB 2 CRCT)

| **Study** | | **Randomization process** | | | | **Timing of identification or recruitment of participants** | | | | **Deviations from the intended interventions** | | | | | | | | | | **Missing outcome data** | | | | | | | **Measurement of the outcome** | | | | | | **Selection of the reported result** | | | | **Overall risk-of-bias judgement** |
| --- | --- | --- | --- | --- | --- | --- | --- | --- | --- | --- | --- | --- | --- | --- | --- | --- | --- | --- | --- | --- | --- | --- | --- | --- | --- | --- | --- | --- | --- | --- | --- | --- | --- | --- | --- | --- | --- |
| **First author, Year** | **Study design** | **Allocation sequence** | **Allocation concealment** | **Baseline difference between groups** | **Risk-of-bias judgement** | **Participants identified before randomisation** | **Selection of individual participants affected by knowledge of the intervention** | **Baseline imbalances suggest differential identification or recruitment of individual participants between intervention groups** | **Risk-of-bias judgement** | **Participants aware that they were in a trial** | **Participant blinding** | **Personnel blinding** | **Protocol deviations** | **Protocol deviations impact on outcome** | **Protocol deviations balanced between groups** | **Appropriate analysis** | **Impact of non-intention to treat analysis** | **Risk-of-bias judgement** | **Outcome data available for nearly all participants** | | **Outcome data available for nearly all participants within a cluster** | **Result not biased by mussing outcome data** | **Missingness dependent on true value** | **Likely that missingness dependent on true value** | **Risk-of-bias judgement** | **Outcome assessment appropriate** | | **Outcome measurement differed between groups** | **Outcome assessors aware of participant intervention** | **Outcome influenced by knowledge of intervention received** | **Assessment of outcome influenced by knowledge of intervention** | **Risk-of-bias judgement** | **Follow a pre-specified analysis plan** | **Selective reporting of outcome within the outcome domain** | **Selective reporting of outcome based on multiple analyses of data** | **Risk-of-bias judgement** |  |
| **Lombard 2016** | CRCT | Yes | Yes | No | **Low risk** | PN | PN | No | **Low risk** | Yes | No | No | PN | N/A | N/A | Yes | N/A | **Low risk** | Yes | | No | No | PN | PN | **Low risk** | No | | PN | No | PY | PN | **Low risk** | Yes | No | No | **Low risk** | **Low risk** |

N/A, not applicable; NI, no information; PN, probably no; PY, probably yes; CRCT, cluster randomised controlled trial

**Additional File 3c** Risk of Bias of non-randomised trials (n=4) using the Risk Of Bias In Non-randomized Studies of Interventions (ROBINS-I) assessment tool

| **Study** | | | **Bias due to confounding** | | | | | | | | | **Bias in selection of participants into the study** | | | | | | **Bias in classification of interventions** | | | | **Bias due to deviations from intended interventions** | | | | | | | **Bias due to missing data** | | | | | | **Bias in measurement of outcomes** | | | | | **Bias in selection of the reported result** | | | | **Overall risk-of-bias judgement** |
| --- | --- | --- | --- | --- | --- | --- | --- | --- | --- | --- | --- | --- | --- | --- | --- | --- | --- | --- | --- | --- | --- | --- | --- | --- | --- | --- | --- | --- | --- | --- | --- | --- | --- | --- | --- | --- | --- | --- | --- | --- | --- | --- | --- | --- |
| **First author, Year,** | **Study design** | Confounding of the effect of intervention | | Analysis based on splitting participants’ follow up time according to intervention received | Intervention discontinuations related to factors that are prognostic for the outcome | Appropriate analysis | Confounding domains controlled for measured validly and reliably | Control for post intervention variables | Appropriate analysis method that adjusted for all the important confounding domains | Confounding domains adjusted for measured validly and reliably | **Risk-of-bias judgement** | Selection of participants based on participant characteristics | Post-intervention variables that influenced selection likely to be associated with intervention | Post-intervention variables that influenced selection likely to be influenced by the outcome | Start of follow-up and start of intervention coincide for most participants | Adjustment techniques used that are likely to correct for the presence of selection biases | **Risk-of-bias judgement** | Intervention groups clearly defined | Information used to define intervention groups recorded at the start of the intervention | Classification of intervention status affected by knowledge of the outcome | **Risk-of-bias judgement** | Deviations beyond what would be expected in usual practice | Deviations unbalanced between groups and likely to have affected the outcome | Important co-interventions balanced across intervention groups | Intervention implemented successfully for most participant | Participants adhere to the assigned intervention | Appropriate analysis used to estimate the effect of starting and adhering to the intervention | **Risk-of-bias judgement** | Outcome data available for nearly all participants | Participants excluded due to missing data on intervention status | Participants excluded due to missing data on other variables needed for the analysis | Proportion of participants and reasons for missing data similar across interventions | Results were robust to the presence of missing data | **Risk-of-bias judgement** | Outcome measure influenced by knowledge of the intervention received | Outcome assessors aware of the intervention received by study participants | Methods of outcome assessment comparable across intervention groups | Systematic errors in measurement of the outcome related to intervention received | **Risk-of-bias judgement** | Selective reporting of outcome within the outcome domain | Selective reporting of outcome based on multiple analyses of data | Selective reporting of outcome based on selected sub-groups | **Risk-of-bias judgement** |  |
| **Cantisano 2022** | Controlled before-after (1 group) | PY | | N | N/A | N | N/A | N | N | N | **SR** | Y | Y | PN | Y | N | **SR** | Y | Y | Y | **MR** | NI | N/A | Y | Y | PY | N | **LR** | N | N | Y | N | PY | **MR** | Y | NI | PY | PN | **MR** | PN | PN | PN | **LR** | **SR** |
| **Debon 2020** | Controlled before-after (≥2 groups) | PY | | N | N/A | N | N/A | N | N | N | **SR** | NI | N/A | N/A | Y | N | **MR** | Y | Y | N | **LR** | NI | N/A | Y | Y | PY | N | **LR** | N | N | Y | Y | N | **MR** | Y | Y | PY | PN | **MR** | PN | PN | PN | **LR** | **SR** |
| **Gilson 2017** | Controlled before-after (1 group) | PY | | N | N/A | N | N/A | N | N | N | **SR** | Y | Y | PN | Y | N | **SR** | Y | Y | N | **LR** | NI | N/A | Y | Y | PY | N | **LR** | N | N | Y | NI | N | **MR** | Y | NI | PY | PN | **MR** | PN | PN | PN | **LR** | **SR** |
| **Hendrie 2022** | Uncontrolled before-after (1 group) | PY | | N | N/A | N | N/A | N | N | N | **SR** | N | N/A | N/A | Y | N | **LR** | Y | Y | N | **LR** | NI | N/A | Y | Y | PY | N | **LR** | N | N | Y | N | PY | **MR** | Y | NI | PY | PN | **MR** | PN | PN | PN | **LR** | **SR** |
| **Perez-Junkera 2022** | Controlled before-after (1 group) | PY | | N | N/A | N | N/A | N | N | N | **SR** | NI | N/A | N/A | Y | N | **MR** | Y | Y | Y | **MR** | NI | N/A | Y | Y | PY | N | **LR** | N | N | Y | N | PY | **MR** | Y | NI | PY | PN | **MR** | PN | PN | PN | **LR** | **SR** |
| **Plaete 2015** | Controlled before-after (≥2 groups) | PY | | N | N/A | Y | Y | Y | Y | Y | **LR** | N | N/A | N/A | Y | N | **LR** | Y | Y | N | **LR** | NI | N/A | Y | Y | PY | N | **LR** | N | N | Y | N | PY | **MR** | Y | NI | PY | PN | **MR** | PN | PN | PN | **LR** | **LR/MR** |
| **Wang 2021** | Controlled before-after (≥2 groups) | PY | | N | N/A | N | N/A | N | N | N | **SR** | Y | Y | PN | Y | No | **SR** | Y | Y | Y | **MR** | NI | N/A | Y | Y | PY | N | **LR** | N | N | Y | N | PY | **MR** | Y | NI | PY | PN | **MR** | PN | PN | PN | **LR** | **SR** |

SR, serious risk; N, No; N/A, not applicable; NI, no information; LR, low risk; PN, probably no; PY, probably yes; Y, yes
